# Iron-dependent degradation of IRP2 requires its C-terminal region and IRP structural integrity

**DOI:** 10.1186/1471-2199-9-15

**Published:** 2008-01-28

**Authors:** Jian Wang, Guohua Chen, Julie Lee, Kostas Pantopoulos

**Affiliations:** 1 Lady Davis Institute for Medical Research, Sir Mortimer B. Davis Jewish General Hospital, Montreal, Quebec, Canada; 2 Department of Medicine, McGill University, Montreal, Quebec, Canada; 3 Department of Biochemistry, University of Texas Southwestern Medical Center, Dallas, Texas, USA; 4 Department of Physiology, University of Texas Southwestern Medical Center, Dallas, Texas, USA

## Abstract

**Background:**

Iron regulatory protein 2 (IRP2), a post-transcriptional regulator of cellular iron metabolism, undergoes iron-dependent degradation via the ubiquitin-proteasome pathway. A stretch of 73 amino acids within the N-terminal domain 1 of the protein was reported to function as an iron sensor. However, mutants lacking this fragment remain sensitive to degradation in iron-replete cells.

**Results:**

To identify elements within IRP2 involved in the control of its stability, we undertook a systematic mutagenesis approach. Truncated versions of IRP2 were expressed in H1299 cells and analyzed for their response to iron. Deletion mutants lacking the entire C-terminal domain 4 (amino acids 719–963) of IRP2 remained stable following iron treatments. Moreover, the replacement of domain 4 of IRP1 with the corresponding region of IRP2 sensitized the chimerical IRP1_1–3_/IRP2_4 _protein to iron-dependent degradation, while the reverse manipulation gave rise to a stable chimerical IRP2_1–3_/IRP1_4 _protein. The deletion of just 26 or 34 C-terminal amino acids stabilized IRP2 against iron. However, the fusion of C-terminal IRP2 fragments to luciferase failed to sensitize the indicator protein for degradation in iron-loaded cells.

**Conclusion:**

Our data suggest that the C-terminus of IRP2 contains elements that are necessary but not sufficient for iron-dependent degradation. The functionality of these elements depends upon the overall IRP structure.

## Background

Iron regulatory proteins, IRP1 and IRP2, post-transcriptionally control the expression of several mRNAs bearing iron responsive elements (IREs). In iron-deficient cells, IRE/IRP interactions account for the stabilization of transferrin receptor 1 (TfR1) mRNA and the translational inhibition of ferritin (H- and L-) mRNAs, resulting in increased uptake and reduced sequestration of iron [1]. IRPs regulate the expression of additional IRE-containing transcripts, such as those encoding erythroid aminolevulinate synthase (ALAS2), mitochondrial aconitase, the iron transporter ferroportin 1, myotonic dystrophy kinase-related Cdc42-binding kinase α (MRCK α), hypoxia inducible factor 2 α (HIF2α), and splice variants of the divalent metal transporter DMT1 and the kinase Cdc14A [2-4]. Experiments with IRP1-/- and IRP2-/- cells and animals revealed that IRP2 exerts a dominant regulatory function *in vivo *[5].

Both IRP1 and IRP2 share significant sequence similarity [1,2,5]. A major difference in their primary structure is that IRP2 contains a unique insertion of 73 amino acids close to its N-terminus (referred to hereafter as 73d). In iron-replete cells, IRP1 binds a cubane 4Fe-4S cluster, which precludes IRE-binding, renders the protein to a cytosolic aconitase and maintains it in a closed conformation [6,7]. Under these conditions, IRP2 undergoes rapid ubiquitination and degradation by the proteasome [1,2,5]. Phosphorylation or defects in Fe-S cluster assembly may also sensitize IRP1 to iron-dependent proteasomal degradation, albeit with slower kinetics compared to IRP2 [8-10].

The mechanism for IRP2 degradation is far from being understood. It has been proposed that the 73d functions as an "iron-dependent degradation domain". One model postulates that the iron-sensing capacity of the 73d is based on site-specific oxidation of conserved cysteine residues upon direct iron binding [11]. Another model suggests that IRP2 degradation is triggered by oxidative modification following high affinity binding of heme within the 73d [12,13]. Nevertheless, experiments in cultured cells showed that IRP2 deletion mutants lacking the entire 73d remain as sensitive to iron as wild type IRP2 [14-16]. Moreover, the 73d failed to destabilize GFP fusion indicator constructs in iron-loaded cells [15], casting further doubt on its proposed function as a necessary and sufficient regulatory element for IRP2 degradation. Recent results showed that 73d is sensitive to proteolytic cleavage and that heme binding only occurs in its truncated form [17].

IRP2 is stabilized in response to hypoxia [14,18,19], by analogy to HIF α subunits that play a crucial role in cellular adaptation to low oxygen levels [20]. Under normoxic conditions, HIF α subunits undergo post-translational modification by the prolyl-hydroxylases PHD1–3, which tag them for ubiquitination by the E3 ubiquitin ligase VHL and degradation by the proteasome [21]. These enzymes, as well as other 2-oxoglutarate-dependent dioxygenases, catalyze the hydroxylation of protein substrates by using 2-oxoglutarate. The reaction yields a hydroxylated amino acid, succinate and carbon dioxide, and proceeds via an iron-oxo intermediate [22]. The availability of ferrous iron, oxygen and ascorbate (presumably to maintain iron in a reduced state) is critical for catalysis.

Experimental evidence supports a mechanism for IRP2 degradation via 2-oxoglutarate-dependent dioxygenases. Thus, dimethyl-oxalyl-glycine (DMOG), a substrate analogue of 2-oxoglutarate, protected IRP2 against iron-dependent degradation [14,15]. Furthermore, ascorbate and other antioxidants accelerated the degradation of IRP2 [15]. Nevertheless, the protective effect of DMOG was restricted to cells that were previously depleted from iron by the chelator desferrioxamine, indicating that the iron-dependent degradation of IRP2 is mediated by multiple mechanisms. One of them appears to interfere with the heme biosynthetic pathway, as succinyl-acetone, an inhibitor of endogenous heme synthesis, stabilized IRP2 against iron [23]; this result has independently been reproduced in various laboratories [13,16,19,24]. The E3 ubiquitin ligases VHL [25] and HOIL-1 [26] are not required for IRP2 degradation in response to iron.

To identify structural elements within IRP2 that may play a role in iron sensing and in the regulation of its stability, we undertook a systematic deletion analysis. Our data suggest that C-terminal sequences are necessary for the iron-dependent degradation of IRP2. However, these sequences only operate in a context of IRP structure and do not have intrinsic iron sensing properties.

## Results

### The C-terminal domain of IRP2 is necessary for its iron-dependent degradation

**Figure 1:**
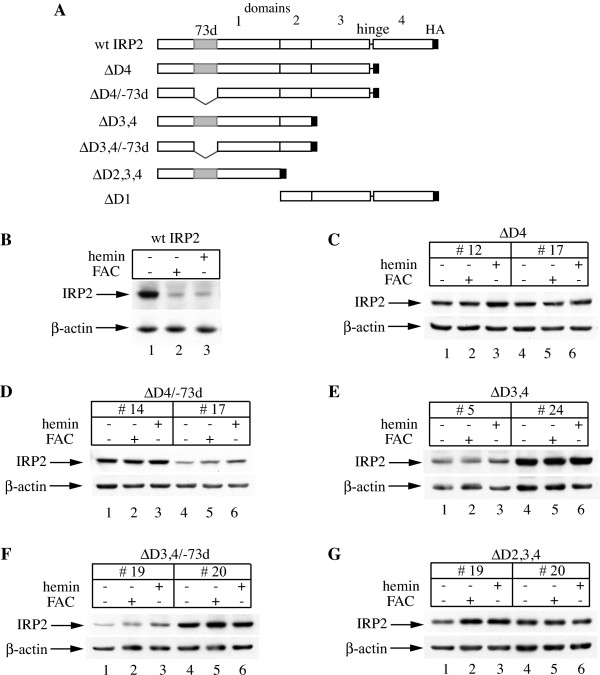
**IRP2 deletion mutants lacking the C-terminal domain 4 are resistant to iron-dependent degradation**. (A) Schematic representation of the deletion mutants showing domains 1–4, the hinge which links domains 3 and 4, and the C-terminal HA tag. The 73 amino acids sequence (73d) within domain 1 is highlighted in gray. (B-G) H1299 cells engineered to express wild type IRP2 or two independent clones of the above mutants (except ΔD1) were treated overnight (14 h) with 100 μM hemin or 30 μg/ml FAC and lysates were subjected to Western blotting with HA (top) and β-actin (bottom) antibodies. No clones expressing ΔD1 could be isolated.

Even though the crystal structure of IRP2 has not yet been solved, on the basis of IRP1 structural data [6,7] and sequence homology between IRP1 and IRP2, it can be predicted that the IRP2 molecule is composed of three compact domains (1–3) linked to a fourth domain via a hinge region (Fig. 1A). Having established that the IRP2-specific 73d is dispensable for iron-dependent degradation, at least in the context of full-length IRP2 and GFP-fusion indicator constructs [14-16], we reasoned that other sequences within IRP2 might possess iron-sensing properties. To address this, we generated a series of IRP2 C-terminal deletion mutants, truncated at domains 2–4 (ΔD2,3,4), 3–4 (ΔD3,4), or 4 (ΔD4), and an N-terminal deletion mutant, truncated at domain 1 (ΔD1). We also generated variants of ΔD3,4 and ΔD4 IRP2 lacking the 73d region (ΔD3,4/-73d and ΔD4/-73d, respectively), to reevaluate a possible regulatory function of this sequence in the deletion mutants. All mutants (Fig. 1A) were transfected into H1299 cells for tetracycline-inducible expression. Several clones of IRP2 C-terminal deletion mutants were isolated; however, no stable clone expressing ΔD1 IRP2 could be obtained. The cells were subjected to treatments with hemin or ferric ammonium citrate (FAC) to analyze the response of the IRP2 deletion mutants to iron. We employed clones with variable expression levels to avoid possible confounding effects due to saturation of the IRP2 degradation machinery [15]. Notably, in contrast to wild type IRP2 (Fig. 1B), iron loading did not affect the expression of any of the truncated IRP2 variants (Fig. 1C–G), even though these treatments are known to promote the degradation of wild type IRP2 [15,16]. These data provide further evidence that the 73d is not crucial for IRP2 degradation. As the minimal truncation of the C-terminal domain 4 appears sufficient for stabilization of the remaining IRP2 polypeptide, these data also suggest that this region may have a functional role in iron sensing.

### The C-terminal domain 4 of IRP2 sensitizes IRP1_1–3_-IRP2_4 _chimeras for iron-dependent degradation

**Figure 2:**
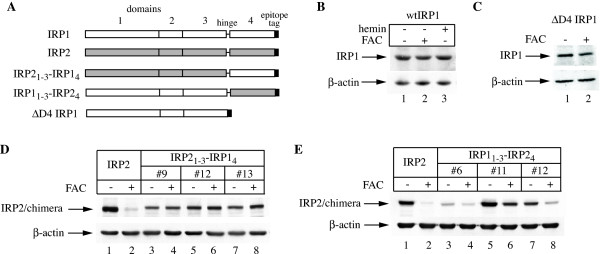
**The C-terminal domain 4 of IRP2 sensitizes IRP1/IRP2 chimeras for iron-dependent degradation**. (A) Schematic representation of wild type IRP1 (white) and IRP2 (grey), the IRP2_1–3_-IRP1_4 _and IRP1_1–3_-IRP2_4 _chimeras and the ΔD4 IRP1 deletion mutant; the IRP1 constructs are tagged with FLAG and the others with HA epitopes. (B-E) H1299 cells engineered to express wild type IRP1, ΔD4 IRP1, wild type IRP2 or the above chimeras (in three independent clones) were treated overnight (14 h) with 30 μg/ml FAC or 100 μM hemin, and lysates were subjected to Western blotting with FLAG or HA (top) and β-actin (bottom) antibodies.

To further evaluate the iron sensing capacity of the IRP2 C-terminus, we generated IRP1/IRP2 chimeras by swapping domains 4 between IRP1 and IRP2 (Fig. 2A). The resulting IRP2_1–3_-IRP1_4 _and IRP1_1–3_-IRP2_4 _constructs were transfected into H1299 cells and at least three clones with variable expression levels were isolated and analyzed for sensitivity to iron. While, as expected, the expression of wild type IRP2 was diminished following treatment with FAC (Fig. 2D, lanes 1–2), the replacement of its domain 4 with that of IRP1 rendered the IRP2_1–3_-IRP1_4 _chimera resistant to iron (lanes 3–8). Furthermore, domain 4 of IRP2 sufficed to decrease the expression of the IRP1_1–3_-IRP2_4 _chimera in iron-loaded cells (Fig. 2E). Under these conditions, wild type IRP1, as well as a ΔD4 IRP1 deletion mutant (lacking the C-terminal domain 4), remained unresponsive to iron treatments (Figs. 2B and 2C, respectively).

**Figure 3:**
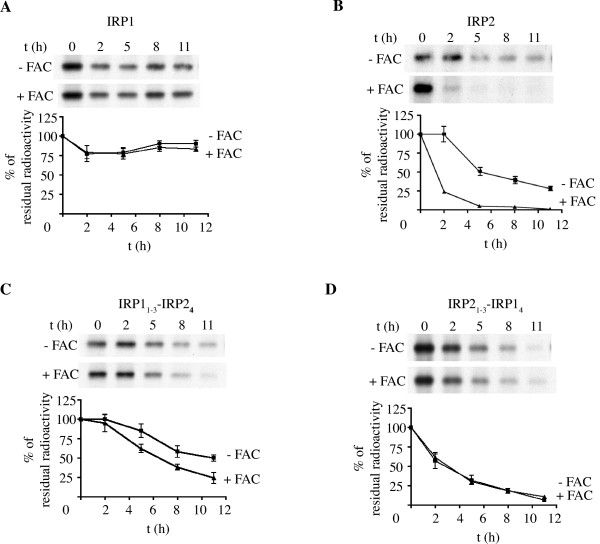
**Pulse chase analysis of the turnover of the IRP1/IRP2 chimeras**. H1299 cells expressing wild type IRP1 (A), wild type IRP2 (B), IRP1_1–3_-IRP2_4 _(C) or IRP2_1–3_-IRP1_4 _(D) were metabolically labeled for 2 h with ^35^S-methionine/cysteine. Subsequently, the cells were chased for the indicated time intervals in cold media in the absence or presence of 30 μg/ml FAC. Cytoplasmic lysates (500 μg) were subjected to quantitative immunoprecipitation with 1 μg HA (Santa Cruz) or FLAG (Sigma) antibodies. Immunoprecipitated proteins were analyzed by SDS-PAGE on a 7.5% gel and visualized by autoradiography. The radioactive bands were quantified by phosphorimaging. The percentage of residual radioactivity from three independent experiments (mean ± SD) is plotted against time.

The half-lives (t_1/2_) of the chimeric proteins were then directly determined by pulse-chase experiments in the absence or presence of FAC and compared to those of wild type IRP1 and IRP2 (Fig. 3). Wild type IRP1 did not decay over 11 h in control or iron-loaded cells (Fig. 3A), consistently with earlier observations [8-10] and references therein]. By contrast, the FAC treatment dramatically reduced the t_1/2 _of wild type IRP2 from ~5 to <2 h (Fig. 3B), again consistently with previous data [15] and references therein]. The presence of IRP2 domain 4 destabilized the IRP1_1–3_-IRP2_4 _chimera compared to wild type IRP1 and, moreover, sensitized it to iron. Thus, the FAC treatment further reduced its t_1/2 _from ~11 to ~6.5 h (Fig. 3C); this effect was statistically significant (p < 0.05). On the other hand, domain 4 of IRP1 abolished the responsiveness of the IRP2_1–3_-IRP1_4 _chimera to iron and apparently accelerated its decay (t_1/2 _~ 3 h) compared to wild type IRP2 (Fig. 3D), possibly by dramatically altering its overall structure. Taken together, these results substantiate the necessity of IRP2 domain 4 in iron sensing and in the targeting of the protein for degradation.

### The swapping of domains 4 between IRP1 and IRP2 impinges on the IRE-binding properties of the chimeras

**Figure 4:**
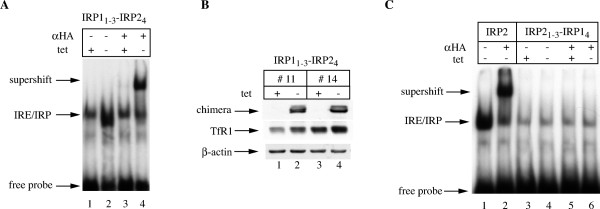
**Functional analysis of the IRP1/IRP2 chimeras**. H1299 cells expressing wild type IRP2, IRP1_1–3_-IRP2_4 _or IRP2_1–3_-IRP1_4 _were grown for 48 h with 2 μg/ml (+) or without (-) tetracycline. (A and C) Cytoplasmic extracts were analyzed for IRE-binding activity with a ^32^P-labeled IRE probe in the absence or presence of 0.2 μg purified polyclonal HA antibody. The positions of the IRE/IRP complexes, the HA-supershifts and excess free IRE probe are indicated by arrows. (B) Analysis of TfR1 expression in two clones expressing IRP1_1–3_-IRP2_4_. Lysates were subjected to Western blotting with HA (top), TfR1 (middle) and β-actin (bottom) antibodies.

Considering that the binding of RNA profoundly alters the conformation of IRP1 [7] and this could potentially affect protein stability, we analyzed the IRE-binding properties of the chimeras by EMSA. The removal of tetracycline from cells expressing IRP1_1–3_-IRP2_4 _induced IRE/IRP complex formation (Fig. 4A, lanes 1–2), indicating that this chimerical protein retains IRE-binding activity. This was verified by supershifting IRE/IRP1_1–3_-IRP2_4 _complexes with the HA antibody (lanes 3–4). The functional implications of this interaction are illustrated in the increased TfR1 levels in cells expressing IRP1_1–3_-IRP2_4 _(Fig. 4B). Interestingly, the replacement of IRP2 domain 4 with that of IRP1 abrogated the IRE-binding capacity of the protein; thus, the IRP2_1–3_-IRP1_4 _chimera was inactive in the binding assay and only IRE complexes with wild type IRP2 could be supershifted with the HA antibody (Fig. 4C). This is in agreement with the possibility that this chimera may possess a distorted structure. As expected, IRP2_1–3_-IRP1_4 _failed to stimulate the expression of TfR1 (data not shown).

### Truncation of the C-terminal domain 4 of IRP2 to identify minimal iron-sensing sequences

**Figure 5:**
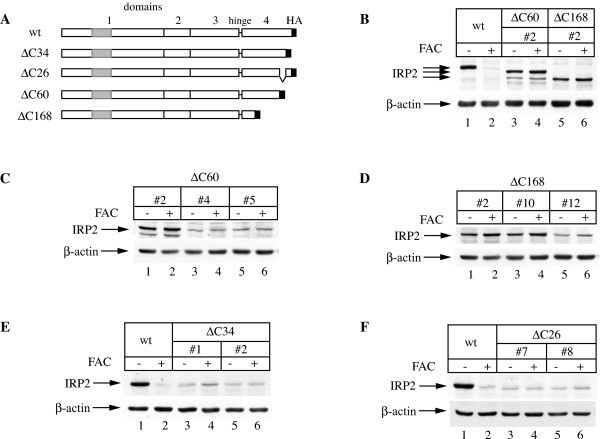
**Truncation of the C-terminal domain 4 stabilizes IRP2 against iron**. (A) Schematic representation of the deletion mutants showing domains 1–4, the hinge which links domains 3 and 4, and the C-terminal HA tag. (B-F) H1299 cells engineered to express the above mutants (in two or three independent clones) were treated overnight (14 h) with 30 μg/ml FAC and lysates were subjected to Western blotting with HA (top) and β-actin (bottom) antibodies. The different migration of wild type IRP2 and the ΔC60 and ΔC168 deletion mutants is illustrated in (B).

The above data are consistent with an involvement of IRP2 domain 4 in tagging the protein for proteasomal degradation. To identify minimal requirements for this activity, further deletion mutants within domain 4 were generated, expressed in H1299 cells and analyzed for their response to iron (Fig. 5). We first noticed that the ΔC168, but also the ΔC60 deletion mutants remained stable in iron-loaded cells (Figs. 5B–D), indicating that the sequence encompassing the 60 C-terminal amino acids of IRP2 may possess iron-sensing properties. To further address this, we generated IRP2 variants lacking either the 34 C-terminal (ΔC34) or the adjacent 26 amino acids (ΔC26). Both the ΔC34, as well as the ΔC26 deletion mutants were resistant to iron-mediated degradation (Figs. 5E and 5F).

Even though the peptides encompassing the 60 C-terminal amino acids of IRP1 and IRP2 are largely conserved (Additional File 1A), they display notable differences and cluster separately in a phylogenetic dendrogram (Additional File 1B). For example, the IRP2 C-terminus is serine-rich, whereas serines are completely absent from the respective segment of mammalian IRP1s. We considered IRP2-specific amino acids in this region as potential regulatory sites and hypothesized that their replacement with IRP1-specific counterparts might confer an IRP1-like phenotype (stabilization in iron-loaded cells). Thus, we focused on non-conserved amino acids that differ significantly (in terms of chemical side chains) between IRP2 and IRP1 and generated IRP2 variants with S929D, S939R and L948Y IRP1-like point mutations. However, none of these manipulations resulted in stabilization of the IRP2 mutants against iron (Additional File 1C).

**Figure 6:**
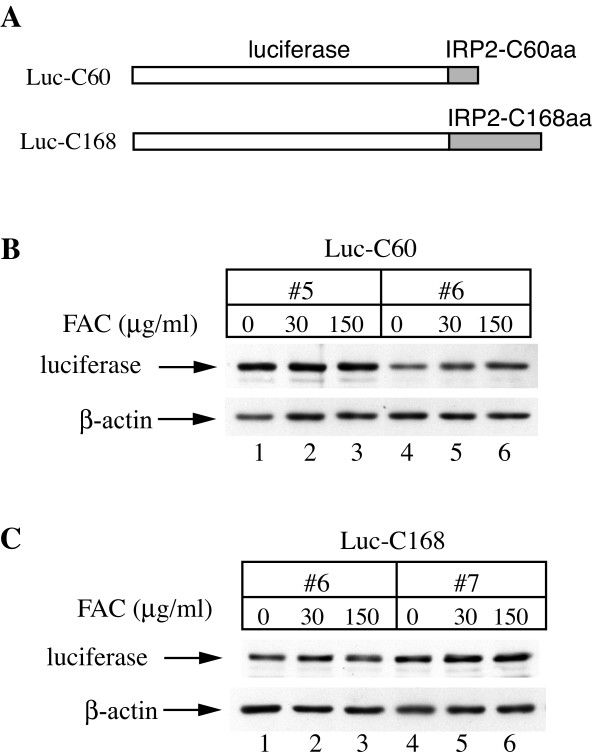
**The C-terminal region of IRP2 encompassing the C60 or C160 amino acids is not sufficient for iron-dependent degradation of a luciferase indicator**. (A) Schematic representation of the luciferase fusion constructs harboring the C60 or C168 peptides of IRP2 (grey) at their C-termini. (B and C) H1299 cells engineered to express these constructs (in two independent clones) were treated overnight (14 h) with the indicated concentrations of FAC and lysates were subjected to Western blotting with luciferase (top) and β-actin (bottom) antibodies.

A *bona fide *iron sensor would be expected to function not only within IRP2, but also in the context of an unrelated protein. To examine this, the region encompassing the C-terminal 60 or 168 amino acids of IRP2 was fused to luciferase indicator constructs (Fig. 6A) and expressed in H1299 cells. However, none of the fusion proteins exhibited any susceptibility to iron loading, and remained stable following treatment of the cells with FAC (Figs. 6B and 6C). In control experiments, we observed that the expression of an unmodified luciferase indicator did not respond to iron (data not shown). Thus, while the C-terminal IRP2 sequences are required for IRP2 degradation, they fail to destabilize the luciferase indicator in response to iron.

## Discussion

The sensitivity of IRP2 to iron has been known for years [27]. Nevertheless, the mechanism for its iron-dependent degradation remains unresolved and, moreover, little progress has been made towards identifying and characterizing IRP2 sequences with iron-sensing properties. The IRP2-specific 73d has been proposed to function as an iron sensor either via direct binding of iron to C168, C174 and C178 [11], or binding of heme to C168 [12] or C201 [13]. Nevertheless, several labs have demonstrated that the above cysteine residues and the entire 73d are dispensable for IRP2 degradation in cultured cells [14,15,19], even though published data with opposing views exist [13]. We reasoned that an unbiased systematic deletion analysis of IRP2 might shed light onto this issue and facilitate the identification of segments of the protein with iron-sensing capacity *in vivo*. To this end, we generated several IRP2 deletion mutants and transfected them into H1299 cells for tetracycline-inducible expression. To avoid saturation effects related to overexpression [15], we selected and analyzed multiple clones with variable IRP2 expression levels for their response to iron treatment. We were unable to isolate stable clones expressing the N-terminal deletion mutant ΔD1; the reason for this is unclear.

The truncation of IRP2 at its C-terminus was sufficient for the stabilization of the protein in iron-loaded cells, independently of the presence or absence of 73d (Fig. 1). These findings not only confirmed that 73d fails to function as an iron sensor *in vivo*, but also shifted our focus for the identification of iron-sensing sequences to the C-terminal domain 4 of IRP2. Further support for the significance of this region in the control of IRP2 stability was provided by experiments with IRP1/IRP2 chimeras, generated by swapping the domains 4 between these proteins (Figs. 2, 3, 4). Thus, domain 4 of IRP2 accelerated the decay of the IRP1_1-3_-IRP2_4 _chimera in iron-loaded cells (Fig. 3C). The stability of ΔD4 IRP1 against iron (Fig. 2C) denotes that this effect is not due to merely the loss of the IRP1 C-terminal region. Along these lines, domain 4 of IRP1 abolished the iron responsiveness of the IRP2_1–3_-IRP1_4 _chimera (Fig. 3D). Collectively, these data suggest that the C-terminus of IRP2 is necessary for its iron-dependent degradation. It should, however, be noticed that the response of the IRP1_1–3_-IRP2_4 _chimera to iron was modest, compared to wild type IRP2 (Fig. 3B), indicating the involvement of additional IRP2 sequences, outside domain 4, in controlling the rate of its degradation.

The iron-sensitive IRP1_1–3_-IRP2_4 _chimera retained RNA-binding activity (Fig. 4A) and was functional in regulating downstream IRE-containing mRNA targets, as shown by the increase in TfR1 expression (Fig. 4B). By contrast, the apparently labile IRP2_1–3_-IRP1_4 _chimera was resistant to iron and failed to bind RNA (Fig. 4C), raising the possibility that the iron responsiveness may be linked to the RNA-binding capacity. We did not directly address this issue; nevertheless, the recent description of other iron-sensitive IRP1/IRP2 chimeras that are inactive in RNA-binding suggests that this is not the case [28]. Considering that the 4Fe-4S cluster is necessary to maintain IRP1 in a compact structure [6,7] and a failure to assemble it sensitizes the protein for slow iron-dependent degradation [8-10], it will be of interest to examine whether the chimerical proteins retain a capacity to assemble a 4Fe-4S cluster, and whether this correlates with their responsiveness to iron.

In an attempt to narrow down the putative iron-sensing region of IRP2, we further truncated domain 4 of the protein and established that the deletion of just small C-terminal segments abolished iron-dependent degradation of IRP2 (Fig. 5). However, the fusion of 60 or 168 C-terminal amino acids of IRP2 to luciferase failed to promote the degradation of this chimerical protein in iron-loaded cells (Fig. 6).

Conceivably, the observed stabilization of the IRP2 deletion mutants is related to a disruption of the overall IRP2 structure that may prevent the exposure of iron-sensing regions. Alternatively, the C-terminal domain of IRP2 may contribute to an iron-dependent rearrangement of the protein structure, which would render it more sensitive to proteolysis. In this scenario, the actual iron sensor could be another factor, such as a proteolytic enzyme, that may attack the destabilized protein upon activation by iron.

## Conclusion

Our results suggest that sequences within the C-terminus of IRP2 are necessary, but not sufficient for iron-dependent degradation, at least outside an IRP context. Thus, the C-terminus of IRP2 does not possess the properties of a *bona fide *iron sensor but its regulatory function requires IRP structural integrity and very likely additional elements within other domains of IRP2 and/or other factors.

## Methods

### Materials

Hemin and ferric ammonium citrate were purchased from Sigma (St. Louis, MI).

### Construction of IRP2 mutants

IRP2 deletion or point mutants were generated from either the pUHD-IRP2 or the pUHD-IRP2_Δ73 _plasmids [15] with the ExSite mutagenesis kit (Stratagene). The forward primer for PCR amplification to yield all C-terminal deletion mutants was 5'-TACCCATACG ATGTTCCAGA TT-3'. The reverse primers were: i) for ΔD4 and ΔD4/-73, 5'-TAAGTCCCAT GGAAACAAAA CT-3'; ii) for ΔD3,4 and ΔD3,4/-73: 5'-TATTGAATTC AGATTAATCT GG-3'; iii) for ΔD2,3,4: 5'-TAAAGTAAGA GAAACTGGCA GA-3'; iv) for ΔC168: 5'-TGTCATTACA GCATCATTAC CT-3'; v) for ΔC60: 5'-GCCCAAGGAA TCTGCATTTT CT-3'; and vi) for ΔC34: 5'-GCTTGTCTGT ATATTCAATG TA-3'. The ΔD1 deletion mutant was generated with 5'-GAGGGATCCA CCATGGGCCC AGAGGTGGTT GGATGT-3' (forward) and 5'-GTGGCACGAA AATTCTCATA CCCATACGAT GTTCCAGATT ACGCTTAGTA AGGATCCTAC G-3' (reverse) primers. The ΔC26 deletion mutant was generated with 5'-ACTGGAAAAG TATTCAGCGT GA-3' (forward) and 5'-GCCCAAGGAA TCTGCATTTT CT-3' (reverse) primers. IRP2 point mutants were generated with following sets of primers: i) for S929D: 5'-AATATACAGA CAGATACTGG AAAAGTA-3' (forward) and 5'-TACTTTTCCA GTATCTGTCT GTATATT-3' (reverse); ii) for S939R: 5'-AGCGTGATTG CTCGATTTGA AGATGAT-3' (forward) and 5'-ATCATCTTCA AATCGAGCAA TCACGCT-3' (reverse); and iii) for L948Y: 5'-TGGAAATAAC ATACTACAAA CATGGA-3' (forward) and 5'-TCCATGTTTG TAGTATGTTA TTTCCA-3' (reverse). To generate the ΔD4 IRP1 deletion mutant, pUHD-IRP1 [29] was digested with MscI/SnaBI (thereby excising domain 4) and religated.

### Construction of IRP1/IRP2 chimeras

A hybrid molecule in which the domain 4 of IRP1 was replaced by that of IRP2 (IRP1_1–3_IRP2_4_) was generated as follows: The region spanning domains 1–3 of IRP1 was PCR-amplified from pUHD-IRP1 [29] with 5'-AGTGTGGGAT CCTGTACAAC CAGG-3' (forward) and 5'-AGTGTGCTCG AGTGCCTGGA TCTCGTCTCT AGT-3' (reverse) primers. Domain 4 of IRP2 was PCR-amplified from pUHD-IRP2 with 5'-AGTGTGCTCG AGGAAGAACA TGTTATA-3' (forward) and 5'-CGTAGGATCC TTACTAAGCG TAATCTGGAA CATCGTATGG GTATGAGAAT TTTCGTGCCA C-3' (reverse) primers. Finally, the two fragments were cloned into the *BamH1 *site of pUHD-IRP1. Likewise, a hybrid molecule in which the domain 4 of IRP2 was replaced by that of IRP1 (IRP2_1–3_IRP1_4_) was generated by PCR-amplification of the region spanning domains 1–3 of IRP2 from pUHD-IRP2 with 5'-GCTCGAGGAT CCCATGGACG CCCC AAA-3' (forward) and 5'-CTGACGCTCG AGTGCATGAA CTTCTTCTCG ACT-3' (reverse) primers. Domain 4 of IRP1 was PCR-amplified from pUHD-IRP1 with 5'-CAGGCACTCG AGCGTCAGTA TGTCATC-3' (forward) and 5'-AGCGTAGGAT CCTTACTAAG CGTAATCTGG AACATCGTAT GGGTACTTGG CCATCTTGCG GAT-3' (reverse), and the two fragments were cloned into the *BamH1 *site of the pUHD10-3 vector [15].

### Construction of luciferase fusion indicators

A common reverse primer was employed to amplify C-terminal sequences of IRP2, including the hemagglutinin (HA) tag and a stop codon, from pUHD-IRP2: 5'-AGTGTGCTCG AGCTAAGCGT AATCTGGAAC-3'. The forward primers were: i) for C168: 5'-AGTGTGGAAT TCAGAGGCAC TTTTGCAAAT-3' and for C60: 5'-AGTGTGGAAT TCCTCTCCGG TAGAGAAACA-3'. The primers introduce *EcoR1 *and *Xho1 *restriction sites. The resulting fragments were subcloned into the respective sites of a firefly luciferase cDNA in the pcDNA3.1 vector.

### Cell culture

H1299 lung cancer cells were maintained in Dulbecco's modified Eagle medium (DMEM) supplemented with 10% fetal bovine serum, 2 mM glutamine, 100 U/ml penicillin, and 0.1 mg/ml streptomycin. Stable H1299 clones for tetracycline-inducible expression of all IRP2 mutants and IRP1/IRP2 chimeras and for non-inducible expression of luciferase fusion constructs were obtained as in [15,29]; the clones expressing wild type IRP1 [8-10] and IRP2 [15] were earlier described. All inducible clones were maintained in DMEM containing 2 μg/ml tetracycline, 2 μg/ml puromycin and 250 μg/ml G418. The cells were plated for 24–48 h in tetracycline-free media to allow expression of transfected proteins. Tetracycline (2 μg/ml) was then added back to shut off transcription of the inducible promoters.

### Western blotting

Cells were washed twice in phosphate-buffered saline (PBS) and lysed in "cytoplasmic lysis buffer" (25 mM Tris-Cl pH 7.4, 40 mM KCl and 1% Triton X-100). Cell debris was cleared by centrifugation and protein concentration was determined with the Bradford reagent (BioRad). Cell lysates (30 μg) were resolved by SDS-PAGE on 8% gels and proteins were transferred onto nitrocellulose filters. The blots were saturated with 10% non-fat milk in PBS and probed with FLAG (M2-FLAG, Sigma), HA (Santa Cruz), TfR1 (Zymed), luciferase (Promega) or β-actin (Sigma) antibodies, diluted 1:1000 (or 1:500 for luciferase) in PBS containing 5% non-fat milk and 0.5% Tween-20 (PBST). Following wash with PBST, the blots with monoclonal FLAG or TfR1 antibodies were incubated with peroxidase-coupled rabbit anti-mouse IgG (1:4000 dilution). The blots with polyclonal HA, β-actin or luciferase antibodies were incubated with peroxidase-coupled goat anti-rabbit IgG or donkey anti-goat IgG (1:5000 dilution). Detection was performed with the enhanced chemiluminescence (ECL) method (Amersham). The immunoreactive bands were quantified by densitometric scanning.

### Pulse chase and immunoprecipitation

Cells were metabolically labeled for 2 h with 50 μCi/ml Trans-[^35^S]-label, a mixture of 70:30 ^35^S-methionine/cysteine (ICN). The radioactive medium was then removed and the cells were chased in cold media. The chase was terminated by wash in PBS. The cells were lysed in a buffer containing 50 mM Tris-Cl pH 7.4, 300 mM NaCl and 1% Triton X-100. Cell debris was cleared by centrifugation and cell lysates were subjected to quantitative immunoprecipitation with the HA or FLAG antibodies. Immunoprecipitated proteins were analyzed by SDS-PAGE. Radioactive bands were visualized by autoradiography and quantified by phosphorimaging.

### Electrophoretic mobility shift assay (EMSA)

Cytoplasmic lysates were analyzed for IRE-binding activity by an electrophoretic mobility shift assay with a ^32^P-labeled IRE probe [30]. Supershifts were performed by the addition of HA antibody to the binding reaction [15].

## Authors' contributions

JW and GC generated and analyzed all IRP2 mutants, JL contributed experimentally, and KP supervised the study and wrote the manuscript. All authors read and approved the final manuscript.

## Supplementary Material

Additional file 1 **Generation and analysis of IRP2 constructs with IRP1-like point mutations at their C-termini**. (A and B) Multiple sequence alignment (A) and phylogenetic dendrogram (B) of the C-terminal C60 amino acids of IRP2 and IRP1, generated by the Clustal W algorithm (MacVector software, version 7.2.3). A value of 0.1 in the dendrogram corresponds to a difference of 10% between two sequences. (C) Analysis of IRP2 constructs bearing S929D, S939R or L948Y point mutations [indicated with asterisk in (A)]. H1299 cells engineered to express these constructs were treated overnight with the indicated concentrations of FAC and lysates were subjected to Western blotting with HA (top) and β-actin (bottom) antibodies. Click here for file
